# Differences in Mean Platelet Volume and Platelet Count between Children with Simple and Complex Febrile Seizures

**Published:** 2017

**Authors:** Ali NIKKHAH, Mohammad Reza SALEHIOMRAN, Seyedeh Samane ASEFI

**Affiliations:** 1 Pediatric Neurology Department, Mazandaran University of Medical Sciences, Sari, Iran.; 2 Pediatric Neurology Department, Non-Communicable Pediatric Diseases Research Center, Amircola’s Children’s Hospital, Babol University of Medical Sciences, Babol, Iran.; 3 Pediatric Department, Amircola’s Children’s Hospital, Babol University of Medical Sciences, Babol, Iran.

**Keywords:** Febrile seizure, Convulsion, Mean platelet volume, Children

## Abstract

**Objective:**

The aim of our study was to find the relationship of MPV (Mean Platelet Volume) levels and platelet counts as markers of inflammation between simple and complex febrile seizures.

**Materials & Methods:**

In this retrospective comparative study, we investigated the recordings of 356 children between 5 months and 6 yr with diagnosis of simple and complex febrile seizure (SFS&CFS) in Amircola’s Children’s Hospital, Babol University of Medical Sciences, Babol, Iran between Mar 2011 and Dec 2015.

**Results:**

Mean age was similar in two groups. The MPV of the CFS group (8.32±0.48fl) was lower than that of the SFS group (8.58±0.34fl) but this difference was not significant statistically. The platelet count of the CFS group (315.03×103 ±117.17×103) was higher than that of SFS group (291.82×103 ± 87.49×103) but there was no significant statistical difference.

**Conclusion:**

We did not find significant differences between two groups. Therefore, further studies about this idea should be performed.

## Introduction

The International League against Epilepsy (ILAE) defined a febrile seizure as “a seizure in association with a febrile illness in the absence of CNS infections or acute electrolyte imbalance in children older than one month of age without previous afebrile seizures”(1). Febrile seizures are most common between 6 months and 5 yr of age, and onset above age 7 is very rare (2, 3). They can be classified as either simple or complex.

A simple febrile seizure (SFS) is brief (<15 min), generalized and once in 24 h. A CFS is focal or secondarily generalized, with duration more than 15 min and more than once in 24 h (4-6). The inheritance model in febrile seizure has not been obvious yet, but some genetic changes in combination of environmental factors are suspected for pathogenesis of this age-limited epileptic syndrome (7).

There are numerous studies on the relationship between iron deficiency anemia and febrile seizure (8, 9). Some but not all of them support the existence of such relationship (10-12). However, there is not any study but one (12) on the relationship between platelet count and MPV levels and febrile seizure types. In that special study, there was a novel idea about relationship between febrile seizure and MPV levels. The authors believed that epilepsy is a brain inflammation disorder and there are more changes that are inflammatory in patients with CFS as a major risk factor for epilepsy versus SFS, therefore, MPV levels decrease in the CFS group compared to the SFS group (12).

In this study, we evaluated MPV levels and platelet counts in children with CFS and SFS in north of Iran.

## Materials & Methods

In this retrospective comparative study, 374 children between 5 months and 6 yr admitted to Amircola’s Children’s Hospital, Babol University of Medical Sciences, Babol, Iran, north of Iran with complaint of seizure and fever (FS) between Mar 2011 and Dec 2015 were enrolled. Eighteen cases with electrolyte imbalance, hypoglycemia, meningitis, encephalitis, shigellosis, Kawasaki disease and severe iron deficiency anemia were excluded from this study. Therefore, 356 children with diagnosis of FS were included.

These patients were divided into unequal two groups: SFS group and CFS group. This classification was performed based on standard definition of SFS and CFS in ILAE (as mentioned previously). The first group (SFS) included 262 children and the second group (CFS) included 94 children. Age, gender, family history of FS and type of FS were recorded for all children. 

The measurements of mean platelet volume (MPV) and platelet count were obtained from the patient’s files. 

The same hospital laboratory expert examined all blood samples.

SPSS 20.0 for Windows (Chicago, IL, USA) was used for analyzing these data. T-test was used for comparing the continuous variables and Chi-square test was used for comparison of categorical variables. P-value of less than 0.05 was considered as significant statistically. 

This study has been done with approval of the Ethics Committee of the Babol University of Medical Sciences. 

Informed consent was taken from all subjects’ parents.

## Results

We evaluated 356 children with FS aged between 5 months and 6 yr. Overall, 262 patients were in SFS group (73.6%) and 94 patients were in CFS group (26.4%). 

Totally, 206 cases were male (160 SFS, 45 CFS) and 150 cases were female (101 SFS, 49 CFS).The mean age of children in SFS group was 35.42±30.56 months. The mean age of children in CFS group was 32.94±24.45 months (Table 1).

The MPV of the CFS group (8.32±0.48fl) was lower than that of the SFS group (8.58±0.34fl), however, this difference was not significant statistically (P=0.071). 

The platelet count of the CFS group (315.03×103 ±117.17×103) was higher than that of SFS group (291.82×103 ±87.49×103) but there was no significant statistical difference (P=0.082) (Table 2). Although, we did not follow this difference and this data was marginal in our study.

**Table 1:** The Demographic Characteristics of Our Patients with FS

	**SFS ( 262)**	**CFS (94)**
**Age (month)**	35.42±30.56	32.94±24.45
**Male**	161	45
**Female**	101	49
**Positive FH** * ** of FS**	80	29
**Negative FH of FS**	182	65

* FH: Familiy History

**Table 2:** Mean Values and Standard Deviations of Platelet and MPV

	**SFS (262)**	**CFS (94)**	***P*** **-value**
**Platelet** *	291.82±87.49	315.03±117.17	0.082
**MPV** **	8.58±0.34	8.26±0.56	0.074

* : ×1000

** : FL (Femtolitre)

## Discussion

Platelets are multifunction blood cells. They play major role in hemostasis but they have other important functions such as inflammatory functions (13). Platelets have a diverse array of receptors that allow them to interact with white blood cells (WBCs), pathogens, tumor necrosis factor (TNF), interleukins (IL-1, IL-6), and inflamed endothelium (14, 15). In an interesting prior study on the use of MPV levels as an inflammatory marker in the differential diagnosis of two febrile seizure types (SFS vs. CFS) (12), we decided to evaluate this role of platelets in our patients in Iran.

FS is classified to two major groups (simple vs. complex FS) based on duration, focal and/or repetition in 24 h. 

In our study, The MPV of the CFS group (8.32±0.48fl) was lower than that of the SFS group (8.58±0.34fl) but this difference was not significant. A significantly lower level of MPV was reported in CFS group versus SFS group (P<0.001) (12). Moreover, the platelet count of the CFS group (315.03×103 ±117.17×103) was higher than that of SFS group (291.82×103 ±87.49×103) but there was no significant statistical difference. In a study, the platelet count of the CFS group was higher than that of SFS group and this difference was significant statistically (P<0.001) (12). Overall, 42 patients were diagnosed with simple febrile convulsions and a control group of 30 normal children were evaluated and level of MPV was statistically significantly high in children with simple FS (16).

There were some limitations in our study compared with similar prior, such as lower cases, especially, small number of cases with CFS. An unusual finding of our study was male-female ratio in CFS group (1.1/1).

Ultimately, we did not find any significant difference of MPV levels and platelet count between SFS and CFS groups.

**In conclusion, **we did not find significant difference between two groups. We believe that more advanced evaluations are needed to marked relationship between inflammatory markers; such as MPV level and seizures (febrile/afebrile).

## References

[1] (1993). Commission on Epidemiology and Prognosis, International League Against Epilepsy Guidelines for epidemiologic studies on epilepsy. Epilepsia.

[2] Shinnar S, Swaiman KF, Ashwal S, Ferriero DM, (2006). Febrile seizure. Pediatric neurology principle & practice.

[3] Shinnar S, Glauser TA, Pellock JM, Bourgeois FD, Edwin Dodson W (2008). Febrile seizures. Pediatric 47 epilepsy, Diagnosis & Therapy.

[4] Lux AL (2010). Treatment of febrile seizures: historical perspective, current opinions, and potential future directions. Brain Dev.

[5] Jons T, Jacobsen SJ (2007). Childhood Febrile Seizures: Overview and Implications. Int J Med Sci.

[6] Iwasaki N, Nakayama J, Hamano K (2002). Molecular genetics of febrile seizures. Symposium I Epilepsia.

[7] Amirsalari S, Keihani doust Z, Ahmadi M (2010). Relationship between iron deficiency anemia and febrile seizures. Iran J Child Neurol.

[8] Ghasemi F, Valizadeh F, Taee N (2014). Iron-deficiency Anemia in Children with Febrile Seizures A Case-Control Study. Iran J Child Neurol.

[9] Bidabadi E, Mashouf M (2009). Association between iron deficiency anemia and first febrile convulsion: a case-control study. Seizure.

[10] Hartfield DS, Tan J, Yager JY (2009). The association between iron deficiency and febrile seizures in childhood. Clin Pediatr.

[11] Ozaydin E, Arhan E, Cetinkaya B (2012). Differences in iron deficiency anemia and mean platelet volume between children with simple and complex febrile seizures. Seizure.

[12] Weyrich AS (2014). Platelets: more than a sack of glue American Society of Hematology (ASH). Hematology.

[13] Rondina MT, Weyrich AS, Zimmerman GA (2013). Platelets as cellular effectors of inflammation in vascular diseases. Circ Res.

[14] Semple JW, Italiano JE Jr, Freedman J (2011). Platelet and the immune continuum. Nat Rev Immunol.

[15] Mahmut Abuhandan, Abdullah Solmaz (2014). Evaluation of Selenium Levels and Mean Platelet Volume in Patients with Simple Febrile Convulsion. Iran J Pediatr.

